# Natural and Engineered Cytokines as Cancer Therapeutics

**DOI:** 10.1146/annurev-cancerbio-070524-040306

**Published:** 2025-10-07

**Authors:** Pilar O’Neal, Sonya Kumar Bharathkar, Amelia C. McCue, Jamie B. Spangler

**Affiliations:** 1 Department of Biomedical Engineering; Translational Tissue Engineering Center, Johns Hopkins University School of Medicine, Baltimore, Maryland, USA; 2 Department of Chemical and Biomolecular Engineering; Bloomberg-Kimmel Institute for Cancer Immunotherapy; Sidney Kimmel Comprehensive Cancer Center, Johns Hopkins University, Baltimore, Maryland, USA; 3 Department of Oncology; Department of Ophthalmology, Johns Hopkins University School of Medicine, Baltimore, Maryland, USA; 4 Department of Molecular Microbiology & Immunology, Johns Hopkins University Bloomberg School of Public Health, Baltimore, Maryland, USA

**Keywords:** cytokines, protein engineering, molecular design, cancer therapeutics

## Abstract

Cytokines represent a diverse group of soluble proteins that play crucial roles in mediating cellular communication in order to regulate cell fate, particularly in the context of the immune system. Because of their critical roles in controlling cell differentiation, proliferation, migration, activation, and survival, cytokines are heavily implicated in the development and progression, as well as in the prevention and clearance, of cancer. Using both native cytokines and engineered versions thereof, ongoing research in the cancer field endeavors to harness the antitumor activities of cytokines to develop targeted immunotherapies. This review surveys the biology of cytokines and their use in cancer treatment, covering several categories of cytokines, including interleukins, interferons, chemokines, growth factors, and hormones. Preclinical and clinical efforts with natural and engineered cytokines along with efforts to combine these molecules with other anticancer modalities are discussed, highlighting both the triumphs and challenges for these essential proteins in oncology applications.

## INTRODUCTION

Cytokines play a crucial role in immune regulation by activating a diverse set of pathways that influence cell behaviors such as differentiation, proliferation, and activation. Due to their vital immune activities, cytokines also play a key role in cancer development and progression, either promoting or inhibiting tumor growth as well as modulating tumor targeting by the immune system. This review covers several categories of cytokines, including interleukins, interferons, chemokines, growth factors, and hormones, with a focus on efforts to leverage the inherent bioactivities of these molecules for cancer immunotherapy. The use of natural cytokines for cancer treatment is discussed, as are emerging approaches to engineer cytokines for improved safety and efficacy. We also address clinical successes and limitations for cytokine therapeutics in oncology to shed further light on the biology of cancer.

## BIOLOGY OF INTERLEUKINS

Interleukins constitute a diverse family of cytokines that modulate immune function, coordinating cellular communication (see Supplemental Figure 1a). Interleukins are most prominently secreted by immune cells, although some are secreted by other cell types. These cytokines regulate diverse biological processes, including inflammation, immune activation, tissue homeostasis, and disease protection or pathogenesis (Yi et al. 2024).

To date, over 40 interleukins have been identified, categorized into four major groups based on structural features: IL-1-like cytokines, class I cytokines [IL-4-like, common gamma (γc)-chain, and IL-6/12-like cytokines], class II cytokines (IL-10-like and IL-28-like cytokines), and IL-17-like cytokines. Notably, some interleukins do not fit into these classifications due to distinct structural properties or a lack of structural data. IL-1-like cytokines are unique from other cytokine families in that they are predominantly composed of β-strands rather than α-helices (Briukhovetska et al. 2021). Class I cytokines adopt a compact fold composed of four α-helices (four-helix bundle) (Wang et al. 2009). Class II cytokines also adopt a helical bundle architecture but consist of six or seven stacked helices. IL-17A and related proteins are distinguished by a cysteine-knot fold (Liu et al. 2013).

Interleukin receptors are multimeric protein complexes embedded in the cell membrane that bind extracellular cytokines to initiate signaling responses through their intracellular domains. Shared receptor usage by multiple cytokines (redundancy) and engagement of multiple receptor types by a single cytokine (pleiotropy) enable flexible albeit complex immune regulation. Many interleukin receptors utilize the Janus kinase-signal transducer and activator of transcription ( JAK-STAT) signaling pathway (Murray 2007), wherein cytokine binding induces receptor homo- or heterodimerization and JAK transphosphorylation, leading to phosphorylation of the receptor intracellular domain and the recruitment, activation, and nuclear translocation of STATs to regulate gene expression.

In the context of cancer, interleukins exhibit dual and often contradictory roles. Given their central role in immune regulation, interleukins have emerged as promising candidates for cancer immunotherapy, offering new approaches for designing monotherapies as well as combination treatments. Interleukin-based treatments, such as IL-2, were among the earliest approved cancer immunotherapies, showing durable responses in 5–10% of patients with metastatic renal cell carcinoma and melanoma (Rosenberg 2012). However, clinical application of IL-2 is limited by toxicity and rapid clearance from serum (Boyman & Sprent 2012). Experience with other natural interleukins has been similarly disappointing, prompting the use of engineering strategies and combination strategies to improve antitumor immune responses.

## BIOLOGY OF INTERFERONS

Interferons constitute a large family of class II cytokines that play major roles in the development and regulation of antiviral or antitumor immunity (Minn 2015) (see Supplemental Figure 1b). Interferons have a conserved layout of six α-helices, and they signal through heterodimeric transmembrane receptors that contain extracellular fibronectin domains, which bind the cytokine.

Interferons fall into three subclasses. Type I interferons, including 12 forms of IFNα along with IFNβ, IFNϵ, IFNκ, and IFN*ω*, are secreted by all nucleated cells and mediate functions such as limiting pathogen spread, increasing antigen presentation, and inducing autophagy (Platanias 2005). Type II interferons contain a single member, IFNγ, which is mainly produced by activated T cells and enhances the function of both innate and adaptive immune cells (Wu et al. 2014). Type III interferons, discovered in the early 2000s, include IFNλ_1_ (IL-29), IFNλ_2_ (IL-28A), IFNλ_3_ (IL-28B), and IFNλ_4_ (Gad et al. 2009). Type III interferons function similarly to type I interferons, although they are less potent and are primarily found near epithelial barriers (Van Winkle et al. 2020). All interferons engage and heterodimerize interferon receptors to initiate JAK-STAT signaling and activate interferon-stimulated genes (Majoros et al. 2017). Because they activate both innate and adaptive immune responses, the antitumor properties of interferons have been explored since the 1960s (Gresser & Bourali 1969). However, toxicities associated with the elevated amounts of interferon needed for tumor inhibition have limited their use. Additionally, interferons can negatively impact antitumor immunity through several mechanisms including upregulation of immunosuppressive molecules and recruitment of immunosuppressive cells into the tumor microenvironment (TME) (Zhang et al. 2021). The context-dependent roles of interferons underscore the need for mechanistic elucidation in order to realize their potential as anticancer drugs.

## BIOLOGY OF CHEMOKINES, GROWTH FACTORS, HORMONES, AND OTHER CYTOKINES

Chemokines are specialized cytokines that orchestrate functions including chemotaxis, hematopoiesis, leukocyte degranulation, and angiogenesis (Moser 2022) (see Supplemental Figure 1c). Chemokines have a conserved tertiary fold, consisting of a three-stranded antiparallel β-sheet followed by an α-helix, and they are categorized into four subfamilies based on the positions of conserved cysteine residues: XC (or C-type), CC, CXC, and CX3C. Signaling is initiated through interaction with seven-pass transmembrane G-protein coupled receptors known as classical chemokine receptors (Hughes & Nibbs 2018). Chemokines are crucial in shaping the function and composition of cells in the TME, and they induce pleiotropic effects that lead to conflicting activities in the context of cancer (Moser 2022).

Other signaling molecules including tumor necrosis factor superfamily (TNFSF) cytokines, growth factors, and hormones also play pivotal immunoregulatory roles in cancer. Apoptosis-inducing TNFSF ligands hold promise in oncology due to their ability to trigger cell death by binding to death receptors (Ababneh et al. 2024). Granulocyte colony stimulating factor (G-CSF), granulocyte macrophage colony stimulating factor (GM-CSF), and thymic peptide hormone thymosin-α1 (Tα1) enhance immune activation and promote antitumor responses, particularly when administered to restore immune cell populations post chemotherapy (Ioannis et al. 2021, Kumar et al. 2022, Wei et al. 2023).

**Figure 1:**
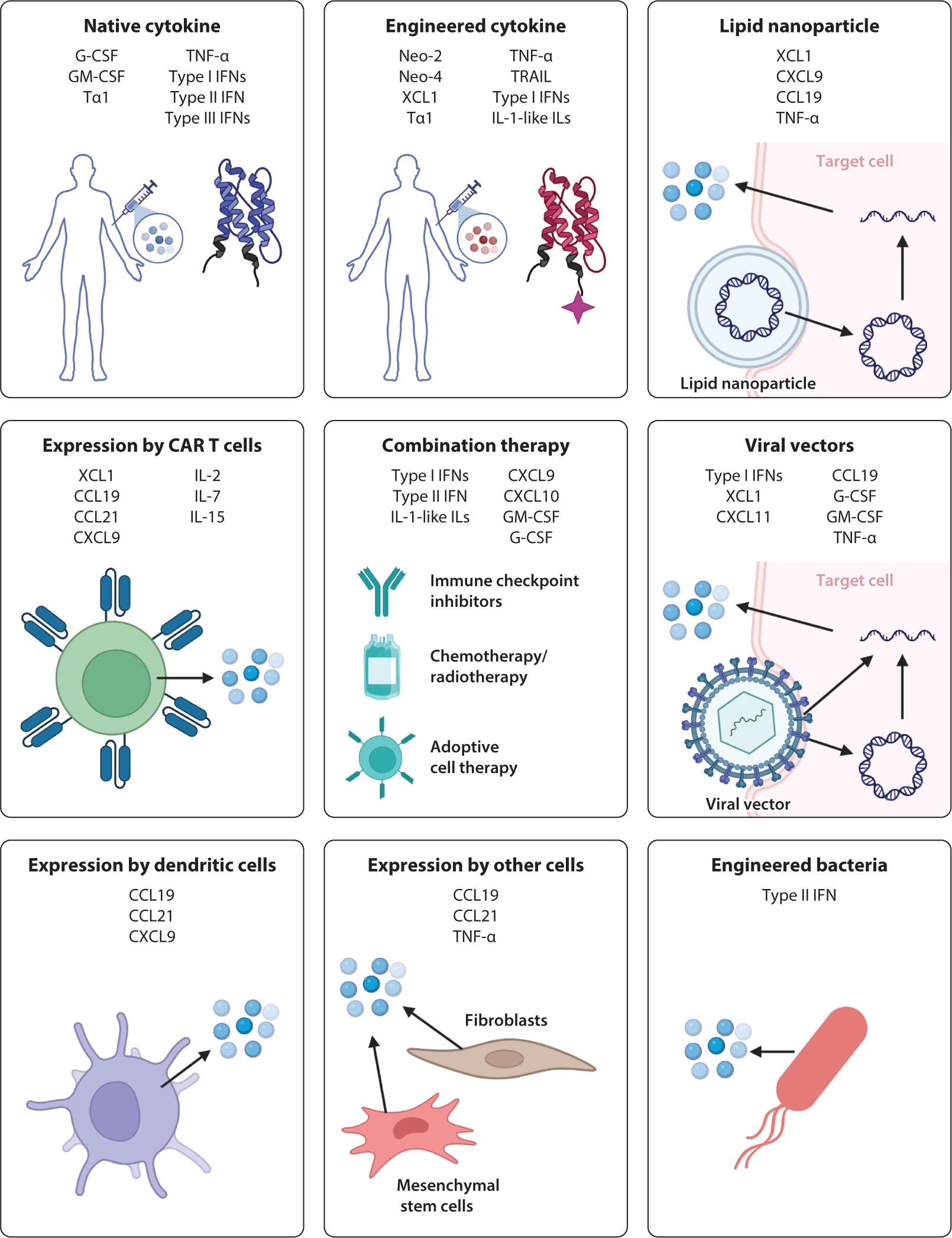
Overview of cytokine administration strategies for cancer therapy. Approaches include the use of native cytokines, engineered cytokines, and cytokine gene delivery via lipid-based nanoparticles or viral vectors. Additionally, cytokines may be expressed by engineered bacteria, CAR T cells, dendritic cells, and a variety of other cell types such as fibroblasts and mesenchymal stem cells. These individual strategies are also often combined with well-established cancer therapy strategies such as immune checkpoint inhibitors, chemotherapy, radiotherapy, and adoptive cell therapy to enhance the efficacy of each individual therapeutic approach. Abbreviations: CAR, chimeric antigen receptor; G-CSF, granulocyte colony stimulating factor; GM-CSF, granulocyte macrophage colony stimulating factor; IFN, interferon; IL, interleukin. Figure created in BioRender; Spangler J. 2025. https://BioRender.com/qbqfgzp.

Overall, this review addresses the current state of cytokine-based cancer therapies, highlighting applications of interleukins, interferons, chemokines, and other cytokine classes. The potential of cytokines as single-agent and combination therapeutics is discussed, with an emphasis on clinical applications (Figure 1). Deepening our understanding of cytokine biology will continue to inform the design of innovative cytokine-based therapies to transform the cancer drug development landscape.

## INTERLEUKIN THERAPIES

### IL-1-Like Cytokines

IL-1-like cytokines (IL-1α, IL-1β, IL-18, IL-33, IL-36α, IL-36β, IL-36γ, IL-1Ra, IL-36Ra, IL-37, and IL-38) play diverse roles in immune regulation and antitumor immunity, with several molecules demonstrating potential as cancer therapeutics. IL-1 family cytokines share a conserved β-trefoil fold, which enables their interaction with IL-1 receptors containing Toll/IL-1 receptor domains to activate the NF-κB signaling pathway.

IL-18 has garnered interest as an immunotherapeutic agent due to its ability to enhance T cell and natural killer (NK) cell activity. Early clinical trials investigating recombinant IL-18 as a monotherapy demonstrated its capacity to increase IFNγ production and bolster cytotoxic immune responses, but efficacy was limited (Robertson et al. 2006). IL-18 has had more success in combination therapies, particularly with immune checkpoint inhibitors (ICIs). Early-phase clinical trials suggest that IL-18 may overcome ICI resistance by reinvigorating exhausted T cells and enhancing effector cell infiltration into tumors (Taheri et al. 2024). Additionally, an engineered IL-18 variant with reduced binding to its natural inhibitor, IL-18 binding protein (IL-18BP), showed promising antitumor efficacy in murine models (T. Zhou et al. 2020).

IL-33, an alarmin released upon cellular stress, drives both pro- and antitumor immune responses. Exogenous IL-33 enhanced antitumor immunity by promoting formation of tertiary lymphoid structures (Amisaki et al. 2025) and also showed synergy with ICIs and chemotherapy in multiple mouse tumor models (Arrizabalaga et al. 2024).

IL-36 cytokines, known for bridging innate and adaptive immunity, have also emerged as promising candidates in cancer immunotherapy. IL-36β and IL-36γ boost CD8^+^ T cell function in preclinical tumor models (Zhao et al. 2019). In combination with doxorubicin treatment, gene delivery of IL-36γ reduced lung metastasis in murine breast cancer models (Chen et al. 2019), and combining intratumoral IL-36γ injection with OX40L and IL-23 significantly enhanced murine colorectal tumor responsiveness to ICIs (Hewitt et al. 2019). Furthermore, secretion of IL-36γ from chimeric antigen receptor (CAR) T cells significantly improved their expansion, persistence, and therapeutic performance (Li et al. 2021).

IL-37, a natural suppressor of inflammation, also exhibits immunomodulatory properties in oncology. Exogenous IL-37 was found to enhance NK cell cytotoxicity against tumor cells and synergize with IL-15 to boost cytokine production (Landolina et al. 2024). Overall, IL-1 family cytokines show promise for cancer therapy either alone or in combination treatments.

### Class I Cytokines (IL-4-Like, γc-Chain, and IL-6/12-Like Cytokines)

Class I cytokines include IL-2, IL-4, IL-6, IL-7, IL-9, IL-11, IL-12, IL-13, IL-15, IL-21, and IL-23. A subset of these, the γc cytokines—IL-2, IL-4, IL-7, IL-9, IL-15, and IL-21—signal through receptor complexes that share the IL-2 receptor gamma (IL-2Rγ) subunit (also called γc or CD132). Due to their potent immunostimulatory effects, γc cytokines have been extensively investigated as cancer therapies, either as recombinantly expressed native proteins or engineered variants thereof.

IL-2 is an essential regulator of lymphocyte function that signals through either a high-affinity (K_D_ ≈ 10 pM) trimeric receptor composed of IL-2Rα (CD25), IL-2Rβ (CD122), and γc or an intermediate-affinity (K_D_ ≈ 1 nM) dimeric receptor composed of only IL-2Rβ and γc. Thus, although it does not contribute to signaling, IL-2Rα expression dictates IL-2 sensitivity. Whereas IL-2Rα is constitutively elevated on regulatory T cells (Tregs), the receptor is sparse on resting immune effector cells (i.e., effector T and NK cells) (Liao et al. 2013, Wang et al. 2005); therefore, IL-2 stimulates immunosuppressive Tregs more potently than proinflammatory immune effector cells, complicating its use in cancer. Nonetheless, IL-2 was the first interleukin-based immunotherapy approved for cancer treatment, with high-dose IL-2 therapy demonstrating durable responses in a subset of patients but also inducing severe toxicities (McDermott et al. 2005). Recent work has explored delivery strategies to enhance the therapeutic index of IL-2, such as liposome-encapsulated mRNA encoding IL-2 ( Jiang et al. 2024). IL-2 has also been combined with other modalities including ICIs (Im et al. 2024).

Other approaches to improve upon IL-2 therapy involve directly engineering the cytokine or designing conjugates/fusion proteins to selectively activate immune effector over Treg cells. One such strategy is the development of IL-2 muteins, which modulate the cytokine’s affinity for its receptor subunits. Cancer therapy efforts have largely focused on reducing affinity for IL-2Rα, which is abundant on Tregs, while also enhancing affinity for IL-2Rβ/γc to potently stimulate immune effector cells (Carmenante et al. 2013). Other strategies include polyethylene glycol (PEG)ylation (Milla et al. 2019), complexation with anti-IL-2 antibodies (Boyman et al. 2006), fusion with IL-2Rα (Ward et al. 2018), and design of immunocytokines that target IL-2 to tumor antigens (Demaria et al. 2024), as reviewed previously (Saxton et al. 2023).

IL-4 promotes tumor progression through immunosuppression and tissue remodeling; thus, anticancer therapies typically antagonize IL-4 or its receptor subunits. However, IL-4 can also exert antitumor effects. For example, a fusion protein consisting of IL-4 fused to an antibody Fc domain has been shown to invigorate terminally exhausted CD8^+^ T cells in the TME (Feng et al. 2024).

IL-7, which signals through a dimeric receptor comprised of IL-7Rα (CD127) and γc, has emerged as a promising candidate for cancer immunotherapy due to its essential role in T cell development, survival, and expansion. IL-7 has been found to boost antitumor immunity in clinical trials, both as a monotherapy (Park et al. 2024) and in combination with IL-12 (Kang et al. 2023). IL-7 has also been integrated into adoptive cell therapies to enhance outcomes (Goto et al. 2021). For instance, a constitutively active IL-7 receptor (denoted C7R), combining the CD34 ectodomain with the transmembrane and intracellular domains of IL-7Rα, promotes T cell survival and antitumor activity (Shum et al. 2017). A phase 1 trial showed that glioblastoma-targeted CAR T cells expressing C7R were well-tolerated and temporarily improved neurologic function (Lin et al. 2024).

IL-15, like IL-2, is essential for lymphocyte activity (Z. Li et al. 2025). IL-15 binds to the IL-2Rβ and γc subunits along with its private IL-15Rα subunit (CD215), typically presented in *trans* by monocytes or dendritic cells (DCs) (Waldmann 2006). Due to their immunostimulatory activities, IL-15 and engineered variants thereof have been explored as cancer therapeutics. IL-15 N72D, a variant with increased affinity for IL-2Rβ and enhanced bioactivity, was combined with a fusion protein comprising the IL-15Rα sushi domain (to facilitate activity) and a human immunoglobulin G1 (IgG1) Fc domain to extend serum half-life (Han et al. 2011). The resulting engineered IL-15 superagonist (nogapendekin alfa inbakicept) was approved by the US Food and Drug Administration (FDA) for invasive bladder cancer. Other engineered IL-15 variants in development include PEGylated IL-15 (Shah et al. 2021), tumor-targeted immunocytokines (Naing et al. 2024), and prodrug forms of IL-15 (Guo et al. 2021). These proteins have been particularly promising in engineered cell therapies, wherein IL-15 enhances cell persistence and function (Ahmed et al. 2025).

IL-21 plays a crucial role in immune modulation, particularly in enhancing T and NK cell activity. While the natural cytokine is well-tolerated in cancer trials (Isvoranu & Chiritoiu-Butnaru 2024), monotherapy responses have been modest; thus, the field has focused on combination therapies with ICIs and other cytokines (https://www.clinicaltrials.gov/ identifier NCT05296772).

Beyond γc cytokines, other class I cytokines also show anticancer potential. These include IL-12 family members, heterodimeric cytokines comprising a four-helix bundle α subunit, and a constitutively associated soluble receptor β subunit. The IL-12 family includes five cytokines, each with distinct α/β subunit pairings: IL-12 (p35/p40), IL-23 (p19/p40), IL-27 (p28/Ebi3), IL-35 (p35/Ebi3), and IL-39 (p19/Ebi3).

IL-12 promotes cytotoxic immune responses, making it a promising cancer immunotherapy candidate. However, systemic delivery causes severe toxicity, limiting clinical use. Numerous preclinical and clinical studies aim to enhance cytokine efficacy while reducing systemic side effects, including nanoparticle-based IL-12 delivery (Li et al. 2020), IL-12-expressing CAR T cells (Lee et al. 2023), and intratumoral administration (Agliardi et al. 2021). IL-12 has also been fused to collagen-binding domains (Momin et al. 2019), extracellular matrix–targeting proteins (Mansurov et al. 2022), and tumor-targeting domains (Curigliano et al. 2024) for localization and retention within the TME. Several clinical trials of IL-12 combination therapies are ongoing, including a phase 2 study of gene-delivered IL-12 (IMNN-001) combined with chemotherapy and an antivascular endothelial growth factor antibody in patients with ovarian cancer (NCT05739981).

IL-27 is a pleiotropic cytokine capable of both stimulating and suppressing immune responses. Native IL-27 demonstrated immune-activating properties in preclinical models but failed in clinical trials as a monotherapy (Mizoguchi et al. 2015). Combination therapies with CAR NK cells are being explored, as IL-27 induces IFNγ production in NK cells (Biggi et al. 2024).

IL-39, the most recently discovered IL-12 family member, has been implicated in modulating immune responses in cancer, although its precise role remains under investigation. Preclinical studies showed that recombinant IL-39 promotes immune cell activation and tumor cell apoptosis, suggesting potential for this molecule as an immunotherapeutic (D’mello et al. 2024).

### Class II Cytokines (IL-10-Like and IL-28-Like)

Class II cytokines (IL-10, IL-19, IL-20, IL-22, IL-24, IL-26, and the IL-28-like family) share a conserved six-helix bundle architecture and signal through heterodimeric receptors composed of class II cytokine receptor subunits.

IL-10 engages a heterotetrameric receptor of two IL-10Rα and two IL-10Rβ subunits. Despite its immunosuppressive properties, IL-10 has shown promise as a cancer therapeutic by enhancing CD8^+^ T cell activity (Oft 2014). PEGylated IL-10 (pegilodecakin) boosted CD8^+^ T cell responses and induced tumor regression in a phase 1 trial (Tannir et al. 2021); however, dose-dependent toxicity has limited clinical translation (Qiao & Fu 2020). Engineering efforts aim to improve specificity and potency. A panel of IL-10 variants with altered IL-10Rβ binding affinities were found to decouple the activities of IL-10, suppressing macrophage activation without stimulating inflammatory CD8^+^ T cells (Saxton et al. 2021). In other work, IL-10 variants with modified IL-10Rβ affinity induced stronger STAT1/STAT3 signaling in monocytes and CD8^+^ T cells, and CAR T cells expanded with these variants demonstrated superior cytolytic function (Gorby et al. 2020).

IL-24 shows potential in cancer therapy, as the native cytokine selectively kills cancer cells (Emdad et al. 2020). Although no successful clinical studies have yet been reported, CAR T cells expressing IL-24 demonstrated improved antitumor activity (K. Zhang et al. 2024a), and an engineered IL-24 variant (M7S, IL-24S) exhibited enhanced secretion and stability, enhancing tumor killing (Pradhan et al. 2022) in preclinical models.

## INTERFERON THERAPIES

The immunostimulatory properties of interferons render them promising candidates for development as cancer drugs. Additionally, the presence of interferons following administration of treatments such as radiation, chemotherapy, and ICIs is positively correlated with successful outcomes, underlining the importance of interferon activity to fight disease (Cheon et al. 2023, Lan et al. 2019, Minn 2015).

### Type I Interferons

Most characterization of interferon use in cancer surrounds type I interferons, particularly IFNα and IFNβ. Type I interferons exert antitumor activity by suppressing neoangiogenesis, inducting cancer cell apoptosis, and increasing tumor antigenicity (Holicek et al. 2024). IFNα became the first FDA-approved cytokine therapy when it was indicated for use in hairy cell leukemia in 1986. It has since been approved to treat chronic myelogenous leukemia, for which it was the first therapy that extended overall survival (Talpaz et al. 2013). However, because of its short half-life and severe toxicity, IFNα is rarely used now in cancer therapy (Aricò et al. 2019).

To overcome half-life limitations, interferons have been conjugated to PEG or fused with other proteins. PEGylated IFNα was evaluated for cancer treatment, although its use has been discontinued (Gosmann et al. 2023, Herndon et al. 2012). Albumin-fused IFNα was also evaluated in cancer therapy, but its efficacy is limited by low retention of cytokine activity; thus, alternative conjugation approaches are being explored (Guo et al. 2020, Hu et al. 2015, Huang et al. 2007, Traub et al. 1981). Albumin-fused IFNα proteins have demonstrated efficacy as cancer vaccine adjuvants, boosting antigen-specific CD8^+^ T cells in mouse models (Tseng et al. 2022).

Type I interferons have been employed extensively in combination therapy regimens. IFNα increased objective response rate and overall survival in combination with doxorubicin or docetaxel (Ben Reguiga et al. 2012, Li et al. 2014, Solal-Celigny et al. 1993), and clinical studies in several cancer types have shown the synergistic effects of combining either PEGylated or non-PEGylated versions of IFNα and IFNβ with ICIs (Razaghi et al. 2023, R. Wang et al. 2023, Zhu et al. 2022). Hydrogel incorporation of IFNα and tyrosine kinase inhibitors proved effective in mouse models of renal cell carcinoma (Ueda et al. 2016). In other preclinical studies, Toll-like receptor 7 (TLR7) agonists showed synergy with type I interferons in suppressing melanoma tumor growth (Sanlorenzo et al. 2025).

Type I interferons have also been evaluated and engineered in other formats to improve efficacy. Intracranial IFNβ adeno-associated virus gene therapy extended survival in a glioblastoma mouse model, and combination with the chemotherapeutic temozolomide enhanced performance (GuhaSarkar et al. 2017). ProIFN, a protein comprising IFNα2b fused to one of its receptor subunits via a tumor protease-cleavable linker, augmented antitumor immunity with reduced toxicity compared to native IFNα2b (Cao et al. 2021). Other formulations under development include IFNβ fused to an antiepidermal growth factor receptor antibody (Yang et al. 2014) and IFNα fused to an ICI antibody (Liang et al. 2018). As the first interferons to be used clinically, type I interferons remain the most actively studied interferon class for cancer therapy.

### Type II Interferon

IFNγ, the sole type II interferon, is beneficial in cancer due to its capacity to polarize CD4^+^ T cells toward the Th1 phenotype, activate CD8^+^ T cells and DCs, and inhibit angiogenesis (Briesemeister et al. 2011). IFNγ has shown preclinical promise in combination therapy regimens. The cytokine heightened sensitivity to several chemotherapy drugs in mouse models of prostate cancer (Korentzelos et al. 2022). Additionally, combining IFNγ delivery with endotoxin-fused IL-4 and IFNα increased ovarian cancer cell killing (Green et al. 2019). IFNγ also showed synergy with TNFα (Schmiegel et al. 1988, Shen et al. 2018), endostatin (Liu et al. 2009), and ICIs (Tang et al. 2024). Furthermore, IFNγ improved the antitumor efficacy of attenuated salmonella (Xu et al. 2022), and administration of IFNγ-expressing bacteria enhanced ICI performance (Li et al. 2024). Immunocytokines that fuse a tumor-homing antifibronectin antibody with IFNγ have also shown preclinical promise (Di Nitto et al. 2023).

Despite these encouraging findings, clinical experience with IFNγ has been mixed. Limiting factors include its concurrent antitumor and protumor activities. For example, IFNγ inhibits CD8^+^ T cells by disrupting granzyme release ( Jing et al. 2024) and promoting expression of immunosuppressive molecules (Bellucci et al. 2015). Nonetheless, multiple clinical trials have evaluated IFNγ as a monotherapy and in combination treatments; however, clinical translation has yet to be realized for type II interferon (Giannopoulos et al. 2003, Zibelman et al. 2023).

### Type III Interferons

Their similar activities to type I interferons motivates investigation of type III interferons in cancer therapy (Kotenko & Durbin 2017). Native IFNλs showed preferential disease targeting and inhibited tumor growth in multiple mouse models (Sato et al. 2006, Wang et al. 2024). PEGylated IFNλ_1_ suppressed tumor growth in mouse hepatocellular carcinoma models (Tian et al. 2014). Gene therapy approaches with IFNλ_1_ and IFNλ_2_ have also shown success (Hasegawa et al. 2016, Liu et al. 2009). Moreover, IFNλ_1_ and IFNλ_2_ showed synergy with IFNα in mouse hepatoma models (Lasfar et al. 2011). Collectively, IFNλs show promise in cancer therapy and are actively being explored in this context.

## CHEMOKINE THERAPIES

Chemokines are cytokines that orchestrate immune cell trafficking, playing complex and contradicting roles in cancer. Their antitumor activities are evidenced by the observation that disruption in chemokine signaling impairs DC recruitment, antigen presentation, and T cell priming and recruitment (Fares et al. 2019). Accordingly, several chemokines have been explored as cancer therapeutics.

### CC Family

CCL19 and CCL21 are ligands for the chemokine receptor CCR7 that serve as potent chemoattractants for DCs, T cells, and B cells and facilitate formation of secondary and tertiary lymphoid structures (Förster et al. 2008). Deficiency of CCL19/CCL21 has been linked to impaired antitumor T cell responses in mice, motivating their use as cancer therapeutics (Marsland et al. 2005). Native CCL19 and CCL21 have shown promising preclinical anticancer effects in various formats, including recombinant proteins (Lu et al. 2015, R. Zhou et al. 2020), encapsulated gene fragments (Liu et al. 2019), secretions from DCs (Salehi-Rad et al. 2023, Wu et al. 2023), CAR T cells (Foeng et al. 2022, Goto et al. 2021, Hu et al. 2022, Pang et al. 2021), fibroblasts (Gray et al. 2018, Iida et al. 2020, Y. Zhang et al. 2024), and oncolytic vaccinia viruses (Li et al. 2012). Additionally, combining CCL19/CCL21 with ICIs synergistically enhanced antitumor activity in multiple mouse models (Chen et al. 2021, Wu et al. 2023, Xu et al. 2024). CCL19 has not been tested in patients, but intratumoral injection of CCL21-expressing autologous DCs led to immune enhancement and tumor shrinkage in a phase 1 clinical trial (Oh et al. 2025, Sharma et al. 2013).

### CXC Family

CXCL9, CXCL10, and CXCL11 bind to the CXCR3 receptor and contribute to antitumor activity through immune cell recruitment (Tokunaga et al. 2018). Increased levels of CXCR3 ligands correlate with stronger T cell presence in tumors (X. Wang et al. 2022), motivating investigation of these ligands as therapeutics. CXCL9 enhanced ICI treatment efficacy in murine ovarian cancer models (Seitz et al. 2022), and gene delivery of both CXCL9 and IL-12 stimulated robust antitumor immunity in mouse colorectal carcinoma models (Lee et al. 2022). Moreover, CXCL9 secretion improved CAR T cell performance in mouse lung cancer models (Tian et al. 2022). CXCL10 has shown preclinical and clinical success (Reschke et al. 2021, Song et al. 2021), including a combination study with ICIs in patients with lung cancer (Lim et al. 2024). Preclinical promise was also seen for CXCL11-armed oncolytic adenoviruses and vaccinia virus (Liu et al. 2016, G. Wang et al. 2023).

CXCL16 is a CXCR6 binding chemokine that plays complex roles in cancer. CXCL16 is expressed by immune and epithelial cells and acts as a chemoattractant and activator of lymphocytes. CXCL16 enhanced antitumor T cell activity in animal models of pancreatic, ovarian, and lung cancer (Mabrouk et al. 2022). However, the chemokine also exhibits protumoral effects, limiting its direct use. Recently, engineered *Escherichia coli* expressing an activating CXCL16 mutant (CXCL16-K42A) led to recruitment and activation of immune cells in mouse cancer models (Savage et al. 2023), presenting a novel approach to achieve antitumor activity with CXCL16.

### CX3C Family

CX3CL1 (fractalkine) binds the receptor CX3CR1 and regulates immune cell recruitment and survival (Ferretti et al. 2014). Increased CX3CL1 expression in tumors has been linked to improved prognosis through lymphocyte recruitment (Sun 2023), supporting its use as an anticancer agent. In mouse breast cancer models, CX3CL1 overexpression inhibited tumor growth and metastasis and showed synergy with an antihuman epidermal growth factor receptor 2 (HER2) antibody (Dreyer et al. 2021). Additionally, administration of CX3CL1 led to improved survival in combination with chemotherapy in a murine fibrosarcoma model (Naessens et al. 2024). The translational potential of CX3CL1 has yet to be explored clinically.

### XC Family

The XCL1–XCR1 axis is uniquely involved in the recruitment of conventional DC type 1 (cDC1) to prime antitumor T cell responses (Syed et al. 2025). However, XCL1 is naturally unstable since it lacks one of two conserved disulfide bonds in chemokines. To circumvent this problem, a stable and highly active form of XCL1 with a second disulfide bond was created that enhanced cDC1 recruitment in mouse models, boosting antitumor immunity and extending survival, especially when combined with ICIs and vaccines (Kamei et al. 2020, 2024; Matsuo et al. 2018, 2021). In other preclinical models, XCL1 expression by CAR T cells (X.-N. Li et al. 2025), oncolytic viruses, and other cell-based vaccines (Chen et al. 2020, Sánchez-Paulete et al. 2018) led to potent anti-tumor activities. Additionally, administration of tumor antigen–derived peptide vaccines fused to XCL1 resulted in tumor suppression, which synergized with ICI or chemotherapy (Chen et al. 2020, Sánchez-Paulete et al. 2018, K. Zhang et al. 2024b). These promising findings motivate the continued therapeutic investigation of XCL1.

## GROWTH FACTOR AND HORMONE THERAPIES

Growth factors and hormones are notorious for their protumorigenic roles, advancing tumor progression, invasion, angiogenesis, and metastasis; thus, most cancer treatments aim to inhibit these ligands or their respective receptors (Lappano et al. 2022). However, some growth factors, such as G-CSF and GM-CSF, and Tα1 have been used to enhance anticancer immunity.

### G-CSF

G-CSF is an essential factor for hematopoiesis, regulating neutrophil production, maturation, and migration. Recombinant human G-CSF (rhG-CSF, filgrastim) was clinically approved in 1991 to treat neutropenia (Fosså et al. 1998) and is used to treat this condition in patients undergoing chemotherapy. Recently, PEGylated rhG-CSF was shown to be more effective than rhG-CSF in breast cancer patients (Huang et al. 2023). Prophylactic G-CSF treatment has also been successfully implemented to prevent chemotherapy-induced neutropenia in breast cancer patients (Blayney et al. 2022). Preclinical studies demonstrated potential for combining G-CSF with oncolytic virotherapy in osteosarcoma (Morales-Molina et al. 2021). However, the tumor-promoting properties of G-CSF have prevented its translation as an anticancer drug.

### GM-CSF

More robust clinical success has been observed with GM-CSF, a pleiotropic growth factor best known for its role in myelopoiesis and hematopoietic cell regulation (Kumar et al. 2022). Recombinant human GM-CSF (rhGM-CSF, sargramostim) was approved by the FDA in 1991, primarily indicated for neutropenia (Lazarus et al. 2021). Beyond monotherapy, combination treatment with chemotherapy, immunotherapy, oncolytic viruses, cancer vaccines, and ICIs has been explored for GM-CSF (Kumar et al. 2022). A recent phase 2 neuroblastoma clinical trial showed that GM-CSF enhances the cytotoxic activity of a tumor-targeted antibody (Mora et al. 2024, 2025). GM-CSF is also employed in tumor vaccines, including secretion from a multiple myeloma cell–based vaccine (MM-GVAX), which led to long-term disease control in treated patients (Biavati et al. 2021). Several ongoing clinical trials combine GVAX with immunotherapeutic modalities (NCT03006302, NCT02451982). GM-CSF is also used in combination with oncolytic viruses, as in the recently FDA-approved RP1 (vusolimogene oderparepvec) (Milhem et al. 2022) and T-VEC (talimogene laherparepvec) (Ferrucci et al. 2021), and others in clinical trials, including Pexa-vac (Monge et al. 2023), JX-594 (Toulmonde et al. 2024), ONCOS-102 (Shoushtari et al. 2023), OH2 (Tan et al. 2025), and MEDI5395 (Davar et al. 2024). Overall, GM-CSF has been the most clinically promising growth factor used in cancer immunotherapies, particularly in vaccine and oncolytic virus approaches.

### Thymosin Alpha 1

Tα1 is a peptide hormone that interacts with TLRs to stimulate signaling pathways that enhance NK and T cell activity, balance the CD4^+^/CD8^+^ T cell ratio, and augment class I major histocompatibility complex (MHC) expression (Costantini et al. 2019). The immunostimulatory properties of Tα1 have been leveraged for cancer therapy in both preclinical and clinical studies (Mao 2023). Tα1 suppressed tumor growth in a preclinical lung cancer model (Liu & Lu 2023), and Fc-fused Tα1 was effective in murine melanoma and breast cancer models (Wang et al. 2018b). Tα1 was engineered with a C-terminal RGDR sequence to target αvβ3 integrin and neuropilin-1, which are upregulated in tumors, and the resulting molecule showed improved specificity and antitumor efficacy (Peng et al. 2020, Wang et al. 2018a). Recombinant Tα1 (thymalfasin) was clinically approved in 2006 to treat lymphocytopenia in cancer patients (Costantini et al. 2019). Clinical trials have demonstrated successful administration of Tα1 as a monotherapy (Niu et al. 2024, Shi et al. 2024) or in combination with chemotherapy (Sha et al. 2024, Stefanini et al. 1998), radiotherapy (Schulof et al. 1985), chemoradiotherapy (Liu et al. 2022), and IFNα (Salvati et al. 1996). Ongoing trials combine Tα1 with ICIs (NCT05790447, NCT06056804, NCT06573398) and cytokines such as IL-12 (NCT06584006), underscoring the promise of Tα1 in oncology.

### Tumor Necrosis Factor Superfamily

TNFSF consists of 19 ligands and 29 receptors, and ligands including TNF-α, FasL, and TRAIL (Apo2L) show promise in cancer therapy by inducing tumor cell apoptosis through interaction with death domain–containing receptors. The earliest example of using TNFSF ligands in cancer was high-dose administration of TNF-α, which induced tumor cell apoptosis and vascular damage in mice (Carswell et al. 1975). Successful clinical studies led to approval in Europe in 1998, but indications were limited by severe systemic toxicities (van Horssen et al. 2006). An ongoing clinical trial is evaluating the combination of TNF-α and Tα1 with and without chemotherapy (NCT05898451). FasL and TRAIL have shown preclinical promise, exhibiting far less toxicity than TNF-α (Sayers & Murphy 2006). Moreover, TRAIL efficacy was enhanced when combined with ionizing radiation in mouse cancer models (Almasan & Ashkenazi 2003, Chinnaiyan et al. 2000, Gong & Almasan 2000). In phase 1 clinical trials, Zn-bound TRAIL was well-tolerated and showed potential for tumor targeting (Sayers & Murphy 2006). Unfortunately, clinical translation of TNFSF ligands remains limited by their conflicting roles in cancer progression, lack of tumor specificity, and induction of cytokine release syndrome (Ababneh et al. 2024). Cell-based expression, gene delivery, and fusion of TNFSF ligands to tumor-targeted antibodies and peptides (Cai et al. 2008, Suo et al. 2022) aim to overcome these challenges. However, preclinical findings have been conflicting, and therapeutic strategies now focus on anti-TNFSF antibodies (Fischer et al. 2020).

## DISCUSSION

Cytokine therapies have emerged as promising strategies in cancer treatment, offering diverse mechanisms to enhance antitumor immunity, including direct tumor cell targeting, immune cell recruitment, and modulation of immunosuppressive factors. However, their pleiotropic nature also presents challenges, necessitating careful optimization to balance efficacy and toxicity.

Among interleukin cytokines, IL-2 and IL-12 have been most successful in cancer treatment, but their clinical utility has been hampered by systemic toxicities. Engineering strategies, including biased variants and tumor-targeted delivery, aim to mitigate these limitations. Similarly, IL-15 and IL-21 have shown promise in enhancing NK and T cell responses, with an engineered IL-15 now in the clinic. IL-12 family cytokines, particularly IL-27 and IL-39, exhibit dual roles in immune modulation; thus, further study is needed to define optimal applications.

Among interferons, type I interferons have achieved the greatest success, with IFNα approved in multiple cancers. However, performance is limited by short half-life and severe toxicities, and engineering efforts will be required to appropriately tune interferon behavior. Type II and type III interferons show preclinical and clinical promise, but these molecules may also require modification to optimize performance.

The most successful chemokines in clinical studies have been CCL21, CXCL9, and CXCL10, but therapeutic approval has been stalled by their pleiotropic nature and inconsistent performance across cancer types. Among growth factors, G-CSF and GM-CSF are most successful and are widely used to treat chemotherapy-induced neutropenia. Among hormones, Tα1 has demonstrated remarkable versatility and is attractive for combination therapies. Although TNFSF ligands represent some of the most promising antitumor agents, their complex roles in cancer progression have limited their clinical application.

Recognizing the therapeutic deficiencies of natural cytokines, intensive efforts are now dedicated to engineering these proteins, for instance through PEGylation, fusion protein design, functional biasing, tumor-targeting approaches, and novel delivery strategies. Additionally, combining cytokine therapies with other anticancer modalities, such as ICIs, cell therapies, and vaccines, improves efficacy and therapeutic window. Overall, cytokines continue to show promise as powerful weapons in the arsenal for designing effective next-generation cancer therapy regimens.

## Supplementary Material

Supplementary Material
