# Supplementary material for: Natural and Engineered Cytokines as Cancer Therapeutics
Source: Annu Rev Cancer Biol. Author manuscript; Available in PMC 2026 Jul 10. (PMC13348789; doi:10.1146/annurev-cancerbio-070524-040306)
Supplement: Supplementary Material [file NIHMS2187081-supplement-Supplementary_Material.pdf]

Supplemental Figure 1

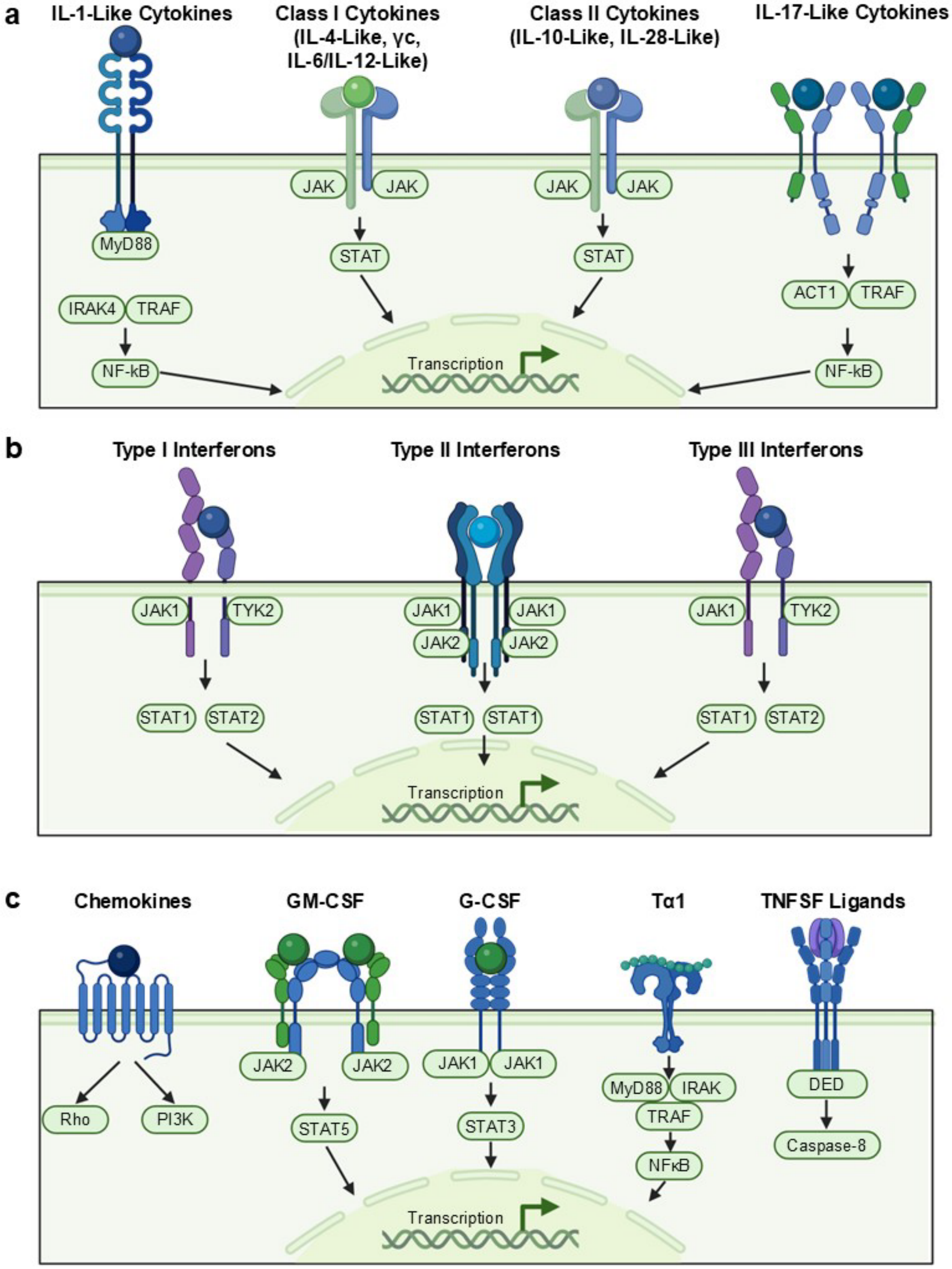

Supplemental Figure 1: Overview of cytokine signaling. **(a)** Representative examples of interleukin ligand/receptor complexes and their downstream signaling mechanisms. IL-1-like receptors engage the MyD88 adaptor protein following ligand binding and recruit IRAK kinases, ultimately activating NF-κB-mediated transcription (*left*). Class I and class II cytokines signal via the JAK/STAT pathway wherein ligand-induced receptor dimerization activates JAKs, leading to STAT phosphorylation and nuclear translocation (*center*). IL-17-like cytokines signal through heterodimeric receptors that recruit the adaptor protein ACT1, leading to activation of NF-κB-mediated transcription (*right*). **(b)** Type I IFNs signal through a heterodimeric receptor composed of IFNAR1 and IFNAR2 (*left*), type II IFN signals through a tetrameric receptor comprised of 2 IFNGR1 subunits and 2 IFNGR2 subunits. (*center*), and type III IFNs signal through a heterodimeric receptor consisting of IFNLR1 and IL-10RB (*right*). All 3 IFN types mediate downstream effects through JAK/STAT signaling. **(c)** Ligand/receptor complexes and signaling pathways for chemokines, GM-CSF, G-CSF, Tα1, and TNFSF ligands (*left to right*). Chemokines signal 7-pass transmembrane chemokine receptors, primarily activating the Rho or Phosphoinositide 3-kinase (PI3K) pathways. GM-CSF and G-CSF use their respective receptor complexes and activate the JAK/STAT pathway. Tα1 binds to various receptors including TLRs, and upon ligand binding, MyD88 is recruited, activating IRAK kinases and downstream NF-κB-mediated transcription. TNFSF ligands bind to their DD-containing TNFSF receptors, resulting in the recruitment of death effector domain (DED)-containing adaptor proteins and the formation of a death-inducing signaling complex (DISC), which subsequently activates caspase-8.

Supplemental Material: Annu. Rev. Cancer Biol. 2026. 10: https://doi.org/10.1146/annurev-cancerbio-070524-040306

Downloaded from www.annualreviews.org. Guest (guest) IP: 162.129.251.72 On: Mon, 29 Jun 2026 22:20:36
